# Multiparametric [^11^C]Acetate positron emission tomography-magnetic resonance imaging in the assessment and staging of prostate cancer

**DOI:** 10.1371/journal.pone.0180790

**Published:** 2017-07-18

**Authors:** Stephan H. Polanec, Piotr Andrzejewski, Pascal A. T. Baltzer, Thomas H. Helbich, Alexander Stiglbauer, Dietmar Georg, Georgios Karanikas, Martin Susani, Wolfgang Wadsak, Markus Margreiter, Markus Mitterhauser, Peter Brader, Katja Pinker

**Affiliations:** 1 Department of Biomedical Imaging and Image-guided Therapy, Division of Molecular and Gender Imaging, Medical University of Vienna, Vienna, Austria; 2 Christian Doppler Laboratory for Medical Radiation Research for Radiation Oncology, Medical University of Vienna, Vienna, Austria; 3 Department of Radiation Oncology, Division of Medical Radiation Physics, Medical University of Vienna, Vienna, Austria; 4 Department of Biomedical Imaging and Image-guided Therapy, Division of Nuclear Medicine, Medical University of Vienna, Vienna, Austria; 5 Clinical Institute of Pathology, Medical University of Vienna, Vienna, Austria; 6 Department of Urology, Medical University of Vienna, Vienna, Austria; Wayne State University, UNITED STATES

## Abstract

**Background:**

The aim of this study was to evaluate whether MP [^11^C]Acetate PET-MRI enables an accurate differentiation of benign and malignant prostate tumors as well as local and distant staging.

**Materials and methods:**

Fifty-six consecutive patients fulfilling the following criteria were included in this IRB-approved prospective study: elevated PSA levels or suspicious findings at digital rectal examination or TRUS; and histopathological verification. All patients underwent MP [^11^C]Acetate PET-MRI of the prostate performed on separate scanners with PET/CT using [^11^C]Acetate and 3T MP MR imaging. Appropriate statistical tests were used to determine diagnostic accuracy, local and distant staging.

**Results:**

MP imaging with two MRI parameters (T2w and DWI) achieved the highest sensitivity, specificity, and diagnostic accuracy of 95%, 68.8%, and 88%, with an AUC of 0.82 for primary PCa detection. Neither assessments with a single parameter (AUC, 0.54–0.79), nor different combinations with up to five parameters (AUC, 0.67–0.79) achieved equally good results. MP [^11^C]Acetate PET-MRI improved local staging with a sensitivity, specificity, and diagnostic accuracy of 100%, 96%, and 97% compared to MRI alone with 72.2%, 100%, and 95.5%. MP [^11^C]Acetate PET-MRI correctly detected osseous and liver metastases in five patients.

**Conclusions:**

MP [^11^C]Acetate PET-MRI merges morphologic with functional information, and allows insights into tumor biology. MP [^11^C]Acetate PET-MRI with two MRI-derived parameters (T2 and DWI) yields the highest diagnostic accuracy. The addition of more parameters does not improve diagnostic accuracy of primary PCa detection. MP [^11^C]Acetate PET-MRI facilitates improved local and distant staging, providing “one-stop” staging in patients with primary PCa, and therefore has the potential to improve therapy.

**Patient summary:**

In this report we investigated MP [^11^C]Acetate PET-MRI for detection, local and distant staging of prostate cancer. We demonstrate that MP [^11^C]Acetate PET-MRI with two MRI-derived parameters (T2 and DWI) achieves the best diagnostic accuracy for primary prostate cancer detection and that MP [^11^C]Acetate PET-MRI enables an improved local and distant staging.

## Introduction

In its multi-step development, cancer has acquired several biological capabilities, which are defined as the hallmarks of cancer [1, 2]. Different imaging modalities can visualize different cancer hallmarks, and their combined application is defined as multiparametric (MP) imaging [3, 4]. In prostate cancer (PCa) MP magnetic resonance imaging (MRI) using different MRI parameters, such as T2-weighted (T2w), dynamic contrast-enhanced (DCE), diffusion-weighted imaging (DWI), and proton magnetic spectroscopic imaging (^1^H-MRSI), is the method of choice for the diagnosis, staging, risk stratification as well as image-guided targeted biopsy of PCa [1]. MP MRI provides both morphological and functional information resulting in a high diagnostic accuracy [2–4]. Positron emission tomography (PET) offers unique functional information and can provide further insights into tumor biology [5]. For imaging, special labeled metabolites that are incorporated into cancer cells can be used to differentiate between cancer and benign tissue. Particularly for PCa, the radiolabeled analogs of the metabolic substrates have been used in the clinical routine: choline; acetate; amino acids and amino acid analogs (e.g., leucine, methionine); and nucleotides. There are promising results with [^11^C] and [^18^F] fluorocholine used as tracers for detecting PCa, which reflect increased choline transport and overexpression of choline kinase in cancer cells [5, 6]. The problem with the radiolabeled metabolites is that they are not specific to PCa, and a higher tracer uptake can be observed at other sites of increased cell metabolism, such as prostatitis, benign tumors, BPH, and non-prostatic malignancies.

Several radiotracers are currently undergoing preclinical or clinical evaluation for prostate cancer imaging to elucidate the underlying processes of tumor development and progression [5]. The radiotracer carbon-11 acetate ([^11^C]Acetate) provides information on the hallmark capability of “reprogramming energy metabolism”. Acetate is one of the central ions in the energy metabolism of human cells and the most common building block for the biosynthesis of fatty acids, which constitute the major components of phospholipids and glycolipids in cell membranes [7]. In human cancer cells, several enzymatic pathways of acetate processing were observed to be upregulated or essential for cell survival [5]. In prostate cancer cells, the upregulation of fatty acid synthetase was found to be the most likely molecular basis for increased cellular uptake. Thus, [^11^C]Acetate PET holds promise to be an additional valuable parameter for prostate tumor evaluation [8–11].

To overcome the limitations of each individual imaging modality, PET/MRI scanners have been developed [12]. Three different fields of applications are feasible with MP PET-MRI: a) morphologic information can be merged with functional information; b) functional imaging parameters can be monitored; and c) molecular and metabolic processes of cancer development can be observed at different levels. To date, the potential of MP PET-MRI in the assessment of prostate tumors has not been investigated in detail.

We hypothesized that, MP [^11^C]Acetate PET-MRI allows a non-invasive quantitative assessment of multiple cancer hallmarks (tumor neoangiogenesis, cell-membrane turnover, fatty acid synthesis, microstructural, and cellular changes), and improves diagnostic accuracy [13]. The aim of this study was to evaluate whether MP [^11^C]Acetate PET-MRI enables an accurate differentiation of benign and malignant prostate tumors as well as local and distant staging.

To reach this aim, we used the currently available MP MR imaging parameters T2-weighted MRI, diffusion-weighted imaging, dynamic contrast-enhanced MRI, and three-dimensional proton MR spectroscopic imaging and the combination of these with the radiotracer [^11^C]Acetate for the assessment of tissue metabolism with PET.

## Materials and methods

This study was approved by the Ethical Committee of the Medical University of Vienna (EK N°1913/2012), written, informed consent was obtained from all patients.

### Patients

Fifty-six consecutive patients (mean age 67y, range 52-83y; PSA level mean 10.4ng/ml, range 2.0–45.5ng/ml) underwent MP [^11^C]Acetate PET-MRI of the prostate. All the patients fulfilled the following inclusion criteria: elevated prostate-specific antigen (PSA) levels >4.0 ng/ml or suspicious findings at digital rectal examination or TRUS; and histopathological evaluation of the suspicious finding. Patients with a history of prostate therapy (e.g., brachytherapy) or therapy to other organs in the vicinity of the prostate, or hormonal therapy, were excluded.

### Imaging

MP [^11^C]Acetate PET-MRI examinations of the prostate were scheduled no longer than two weeks apart (average 2.9d; median 0d; max = 14d). In case of a previous biopsy, the time interval between biopsy and MP [^11^C]Acetate PET-MRI was at least four weeks to avoid artifacts from post interventional hemorrhage.

### Multiparametric MRI

MP MRI examinations were performed on a 3T MRI (MAGNETOM Tim Trio, Siemens Healthcare, Erlangen, Germany) with vendor-supplied combined spine array and body array receive-only coils. No endorectal coil was used. Before imaging, patients underwent an intestinal lavage and were asked to empty the bladder. The patients were positioned in the feet-first supine position. The rectum was filled with ultrasound gel (Ultraschall Gel, Gello GmbH, Germany) to avoid artifacts. To suppress bowel movements, 10mg of Hyoscine butyl-bromide (Buscopan, Boehringer Ingelheim, GmbH, Germany) was applied before the examination. The MRI protocol included the following sequences:

- Anatomical, high-resolution, T2-weighted turbo spin echo in all three planes (TR/TE/TI 4000/101/230ms; field of view [FOV] 200x200mm, 20 slices, slice thickness (SI) 3.0.mm; matrix 320, flip angle 150°, GRAPPA factor 2)
- Diffusion-weighted, single-shot echo-planar imaging with inversion recovery fat suppression (TR/TE 3300/60ms; spectrally adiabatic inversion recovery [SPAIR] fat suppression, FOV 260, 20 slices, SI 3.6 mm; matrix 160; 8 averages, b-values 100, 400 and 800sec/mm^2^; GRAPPA factor 2)
- ^1^H –MRSI: Chemical Shift Imaging (CSI) / Number of Repetitions (NEX) spectroscopy (TR/TE 750/145ms, NEX = 0). The FOV was positioned around the prostate and varied according to the size of the prostate; acquisition time between 8 and 10min.
- Three T1-mapping sequences (TR/TE 3.85/1.42, flip angle 2.5,10, 20 degrees, matrix 256, FOV 260mm, slice thickness 3.6mm, 4 averages), before dynamic scanning, to measure the T1 values with the variable flip-angle method
- Dynamic Contrast-Enhanced (DCE)-MRI, using a view-sharing, three-dimensional, T1-weighted gradient echo sequence (TWIST) (TR/TE 3.85/1.42ms, flip angle 12 degrees, 70 repetitions, k-space subsampling with central region A 30% and sampling density 25%, resulting in a temporal resolution of 4.22s, FOV 260 mm, matrix 160, GRAPPA factor 2)

Gadoteratemeglumine (Gd-DOTA, Dotarem^®^, Guerbet, France) was used as contrast agent for DCE MRI and was injected intravenously as a bolus at 0.2ml/kg body-weight using a power injector at 4ml/s, followed by a 20ml saline flush after three baseline scans. The duration of MP MRI protocol was approximately 45 minutes.

### [^11^C]Acetate PET/CT

All patients underwent whole-body PET/CT scanning using a combined PET/CT in-line system (Biograph 64 TruePoint PET/CT system, Siemens, Erlangen, Germany). Patients were injected intravenously with approximately ~740 MBq to 850MBq of [^11^C]Acetate based on the patient`s bodyweight, and uptake time was 20 minutes. [^11^C]Acetate was prepared in-house using a fully automated radiosynthesizer (GE FASTlab^®^, GE Healthcare, USA) with dedicated software and single-use cassettes produced under good manufacturing practice (GMP).

A supine PET dataset (4–5 bed positions, 3min/ bed position) from the base of the skull to mid-thigh and a low-dose, unenhanced CT scan (spiral scanning; Sl/pitch 3.0/0.55mm, 120kV, 230mAs) for attenuation correction and image registration was obtained. The PET data was reconstructed using the TRUE-X algorithm with four iterations (21 subsets, 168x168 Gaussian filter, FWHM 3mm).

### Data fusion and analysis

PET and MRI datasets were registered and fused using Mirada RTx software (Mirada Medical Ltd, Oxford, UK). Initial registration between all MRI and PET/CT datasets, because the imaging was performed in a common coordinate system, was checked and corrected for potential inter-sequence motion. PET-MRI fusion was performed as follows:

- the high resolution T2-weighted MRI dataset was registered with the CT images (fine, rigid, mutual information-based algorithm followed by manual corrections)
- the computed MRI-CT registration matrix was applied to the PET dataset.

MP [^11^C]Acetate PET-MRI data were evaluated prospectively by an experienced radiologist and a nuclear medicine physician according to the following criteria in consensus. The readers were aware of the PSA levels, but not clinical and histopathology findings.

Additionally, for the patients that underwent a radical prostatectomy, the slides from the whole-mount sections with the delineations made by the pathologist were digitalized using a scanner. Each slice has been assigned to the respective PET-MRI slice and suspicious regions were analyzed against the histopathological gold standard.

### Imaging analyses

All lesions identified on MRI were evaluated according to the ESUR guidelines [14, 15]. On [^11^C]Acetate PET/CT a focally increased tracer uptake was rated positive for malignancy. For multiparametric analyses, combinations of T2w with up to four functional parameters (DWI, ^1^H-MRSI, DCE, [^11^C]Acetate PET) were evaluated. We did not assess combinations of solely functional parameters because neither can be used for diagnosis without the information provided by T2w imaging.

### MRI

In each patient according to ESUR guidelines the most suspicious lesion was defined as the dominant intraprostatic lesion (DIL). Each suspicious lesion was graded according to the PI-RADS classification system version 1 for T2w, DWI, DCE and ^1^H-MRSI [14, 15]. We used PI-RADS version 1 as this includes ^1^H-MRSI in its reporting recommendations. The DIL findings were then dichotomized into benign and malignant. The scores from 1 to 3 on T2w, DWI, and DCE, and from 1 to 2 for ^1^H-MRSI, were rated benign. A DIL was rated positive for malignancy if the score was 4 or 5 on T2w, DWI, and DCE. For MRSI, an elevation of the choline peak (PI-RADS 3 version 1) was already considered indicative of malignancy according to the literature [16].

### [^11^C]Acetate PET/CT

Focally increased [^11^C]Acetate uptake in tumors was quantified by maximum standard uptake values (SUV_max_) normalized to body weight using a MultiModalityWorkstation (MMWP) (Siemens Healthcare). Areas with uptake visually exceeding the mediastinal one were considered suspicious. Tumor to background ratios were defined as the respective SUV (maximum and mean of 1cc around maximum) normalized to the mean SUV measured in the mediastinum. However, no thresholds have been defined for assigning scores 1–5. For SUV_max_ determination, the reader placed a sphere around the lesion. This sphere encompassed the entire lesion, but excluded physiologic [^11^C]Acetate uptake in the surrounding tissue.

### Multiparametric data analyses

For multiparametric analyses, the following combinations of T2w with up to four functional parameters (DWI, ^1^H-MRSI, DCE, [^11^C]Acetate PET) were evaluated.

### Two parameters

Imaging with T2w and one additional functional parameter, i.e., DWI, ^1^H-MRSI, DCE, or [^11^C]Acetate PET, was classified as positive if at least one imaging parameter was indicative of PCa.

### Three parameters

For assessment of MP imaging using T2w and two of the functional imaging parameters (i.e., DWI, ^1^H-MRSI, DCE, [^11^C]Acetate PET), the following reading scheme was used.

1. If all three parameters were positive for malignancy—MP imaging was considered positive for PCa.
2. If all three parameters were negative for malignancy—MP imaging was considered negative for PCa.
3. If two of the three parameters were positive for malignancy, MP imaging was considered positive for PCa.
4. If two of the three parameters were negative for malignancy, MP imaging was considered negative for PCa.

### Four parameters

For the assessment of MP imaging with T2w and three of the functional parameters (i.e., DWI, ^1^H-MRSI, DCE, [^11^C]Acetate PET), the following scheme was used:

1. If all four parameters were positive for malignancy, MP imaging was considered positive for PCa.
2. If all four parameters were negative for malignancy, MP imaging was considered negative for PCa.
3. If three of four parameters were positive for malignancy, MP imaging was considered positive for PCa.
4. If three of four parameters were negative for malignancy, MP imaging was considered negative for PCa.
5. In case of a tie where two of four parameters were positive for malignancy, MP imaging was considered positive for PCa.

### Five parameters

For the assessment with all five imaging parameters acquired (T2w, DCE, DWI, ^1^H-MRSI, and [^11^C]Acetate PET), the following scheme was used.

1. If all five parameters (T2w, DCE, DWI, ^1^H-MRSI, and [^11^C]Acetate PET) were positive for malignancy, MP imaging was considered positive for PCa.
2. If all five parameters (T2w, DCE, DWI, ^1^H-MRSI, and [^11^C]Acetate PET) were negative for malignancy, MP imaging was considered negative for PCa.
3. If three of five parameters were positive for malignancy, MP imaging was considered positive for PCa.
4. If three of five parameters were negative for malignancy, MP imaging was considered negative for PCa.

### Lymph node and distant staging

Lymph nodes (LN) dissected from each anatomic site were separated into four LN regions as follows: right and left external and internal common iliac; right and left fossa obturatoria. The verification was performed on regional and not node-by-node basis. A LN was rated suspicious for metastasis on MP MRI according to the following criteria: short axis diameter > 10mm, round shape, matted fatty hilum, and increased contrast agent uptake. In [^11^C]Acetate PET/CT, LN with a qualitatively increased tracer uptake compared to background activity were considered positive for metastasis. If [^11^C]Acetate PET/CT was positive, MP [^11^C]Acetate PET-MRI was considered positive for LN metastasis.

MP [^11^C]Acetate PET data were evaluated for the presence of distant metastases, (i.e., distant LN [aortic, common iliac, inguinal (deep), inguinal (superficial, femoral), supraclavicular, cervical, scalene, retroperitoneal], as well as skeletal and organ metastases [9].

### Reference standard

Histopathology was defined as the standard of reference. Histopathology specimens of the prostate were obtained by either transrectal ultrasound, MR-guided in-bore biopsy [17] or radical prostatectomy (15/56 patients). In 15 patients undergoing radical prostatectomy a pelvic LN dissection was performed. All distant organ metastases detected by MP [^11^C]Acetate PET-MRI were verified by image-guided biopsy. Patients with benign histopathology results have been actively monitored (digital rectal examinations, PSA measurements) by the urologist and have not shown any signs indicative of prostate cancer.

### Statistical analysis

Statistical analyses were performed using SPSS (SPSS Statistics ver. 22.0, IBM Corp., USA) and Medcalc 15.8 (Medcalc software bvba, Ostend, Belgium). The calculations were performed patient-wise (Obuchowski level 2 analysis—requirement to correctly localize the lesion). Receiver operating characteristics (ROC) were calculated and plotted for the PSA score and all imaging modalities and their combinations. The following parameters were derived from the ROC curves: area under the curve (AUC) (std. error, statistical significance, as well as upper and lower 95%-CI bound); sensitivity; specificity; positive predictive value (PPV); and negative predictive value (NPV).

## Results

Histopathology classified 40/56 (71.4%) as malignant and 16/56 (28.6%) as benign. In 15/40 patients therapy of choice was radical prostatectomy with LN dissection, 19 patients were treated by radiation therapy, and six patients are under active surveillance. The 16 patients with benign histopathology results were monitored actively (PSA measurements, MRI, urologic checkups). During a mean follow up period of 43.5 month (range 25–76 months) no new cancer was detected. Two patients were lost in follow up process. Detailed histopathology results are summarized in Table 1.

**Table 1:** Histopathological characteristics of the dominant intraprostatic lesion and maximum standard uptake values from [^11^C]Acetate.

**Benign results** *N = 16/56* (28.6%)		**Maximum SUV** Mean 4.8	**Minimum ADC** Mean 1004
Prostatitis	*7/16* (43.8%)	4.6	1053
BPH	*9/16* (56.2%)	5.0	888
**Prostate cancer** *N = 40/56* (71.4%)		**Maximum SUV** Mean 4.6	**Minimum ADC** Mean 831
Gleason Score 6 (3+3)	*19/40* (50%)	4.7	841
Gleason Score 7 (4+3)	*13/40* (32.5%)	4.5	855
Gleason Score 8 (4+4)	*2/40* (5%)	6.3	934
Gleason Score 9 (5+4)	*5/40* (10%)	4.5	744
Gleason Score 10 (5+5)	*1/40* (2.5%)	5.9	549

SUVmax for benign lesions ranged from 3.5–8.5 (mean, 4.8) and for malignant lesions from 1.5–9.6 (mean, 4.6). SUVmax for benign and malignant lesions was not significantly different (p>0.05). Minimum ADC value for benign lesions raged from 581–1338 (mean, 1004) and for malignant lesions from 414–1312 (mean, 831). Mean minimum ADC for benign and malignant lesions was significantly different (p<0.05) (cf. Table 1).

Sensitivities, specificities, diagnostic accuracies, and the AUC for the several assessments in MP [^11^C]Acetate PET-MRI are listed in Table 2.

**Table 2:** Sensitivities, specificities, diagnostic accuracy, area under the curve, significance, and 95% confidence intervals for the assessment of each single parameter, and MP [^11^C]Acetate PET-MRI with one to five parameters.

	Sensitivity	Specificity	Accuracy	AUC^6^	*p*^7^	Asymptotic 95% Confidence Interval	
						Lower Bound	Upper Bound
**Single parameter**							
**T2w**^1^	.950	.588	.839	.788	.*001*	.634	.941
**DWI**^2^	.950	.500	.804	.725	.*009*	.559	.891
^**1**^**H-MRSI**^3^	.650	.625	.625	.638	.*110*	.475	.800
**DCE**^4^	.775	.563	.714	.669	.*050*	.504	.833
**PET**^5^	.775	.313	.643	.544	.*612*	.373	.715
**Combination with two parameters**							
**T2w and DWI**	.950	.688	.875	.819	.*000*	.674	.963
**T2w and** ^**1**^**H-MRSI**	.650	.688	.661	.669	.*050*	.511	.827
**T2w and DCE**	.775	.750	.768	.763	.*002*	.618	.907
**T2w and PET**	.750	.688	.714	.719	.*011*	.565	.873
**Combination with three parameters**							
**T2w, DWI and** ^**1**^**H-MRSI**	.950	.625	.857	.788	.*001*	.634	.941
**T2w, DWI and DCE**	.950	.563	.839	.756	.*003*	.596	.917
**T2w, DWI and PET**	.950	.438	.804	.694	.*025*	.523	.864
**Combination with four parameters**							
**T2w, DWI,** ^**1**^**H-MRSIand DCE**	.950	.563	.839	.756	.*003*	.596	.917
**T2w, DWI,** ^**1**^**H-MRSI and PET**	.950	.500	.821	.725	.*009*	.559	.891
**T2w, DWI, DCE and PET**	.950	.563	.839	.756	.*003*	.596	.917
**Combination with five parameters**							
**T2, DWI,** ^**1**^**H-MRSI, DCEand PET**	.900	.563	.804	.731	.*007*	.570	.893

^1^ T2-weighted MRI

^2^ diffusion-weighted imaging

^**3**^ three-dimensional proton MR spectroscopic imaging

^**4**^ dynamic contrast-enhanced MRI

^5^ positron emission tomography

^6^ area under the ROC curve

^7^ significance

**Fig 1:**
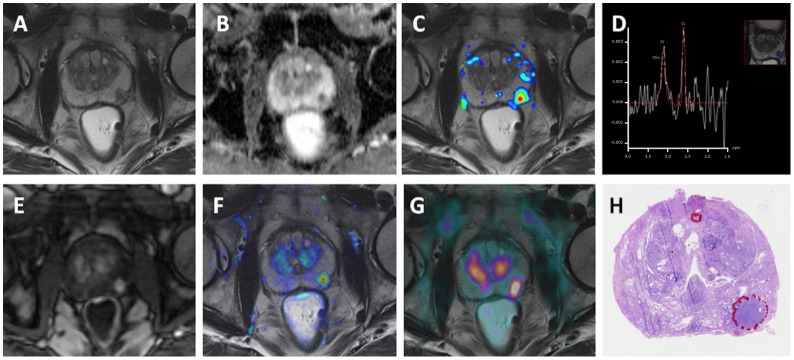
MP [^11^C]Acetate PET-MRI performed in a 68-year-old patient with an elevated prostate-specific antigen (PSA) level (5.3ng/ml) at the time of imaging. (a) Axial 3-mm thick T2-w image (TR/TE/TI 4000/101/230ms) of the middle third of the prostate. The observers described a focal hypointense lesion in the left peripheral zone (T2w-positive). (b) On the ADC map, the lesion presents as a focal area with low signal intensity, with corresponding high signal intensity on b800s/mm2 images (DWI-positive). (c-d) ^1^H-MRSI shows an elevated choline/citrate ratio in the suspicious region (^1^H-MRSI-positive). (e-f) The DCE-MRI shows a focal contrast enhancement for the suspicious area (e –T1w image 80s post contrast, f—K^trans^ map overlaid on T2w image) (DCE-positive). (g) [^11^C]Acetate PET-MRI shows a focal tracer hotspot in this area with a maximal SUV 6.5 (PET-positive). Multiparametric [^11^C]Acetate PET-MRI was rated true-positive in this patient. (h) Histopathological work-up after RPE confirmed a high-grade PCa Gleason 9 (5+4) tumor.

MP imaging with two MRI parameters (T2w and DWI) achieved the highest sensitivity of 95% and a specificity of 68.8%, resulting in a diagnostic accuracy of 88%, with an AUC of 0.82. None of the other assessments, either with a single parameter (AUC, 0.54–0.79), or different combinations with two parameters (AUC, 0.67–0.76), three parameters (AUC, 0.69–0.79), four parameters (AUC, 0.73–0.76), or five parameters (AUC, 0.731), achieved results as good as that (cf. Fig 1, and Supporting information S1 Fig).

**Fig 2:**
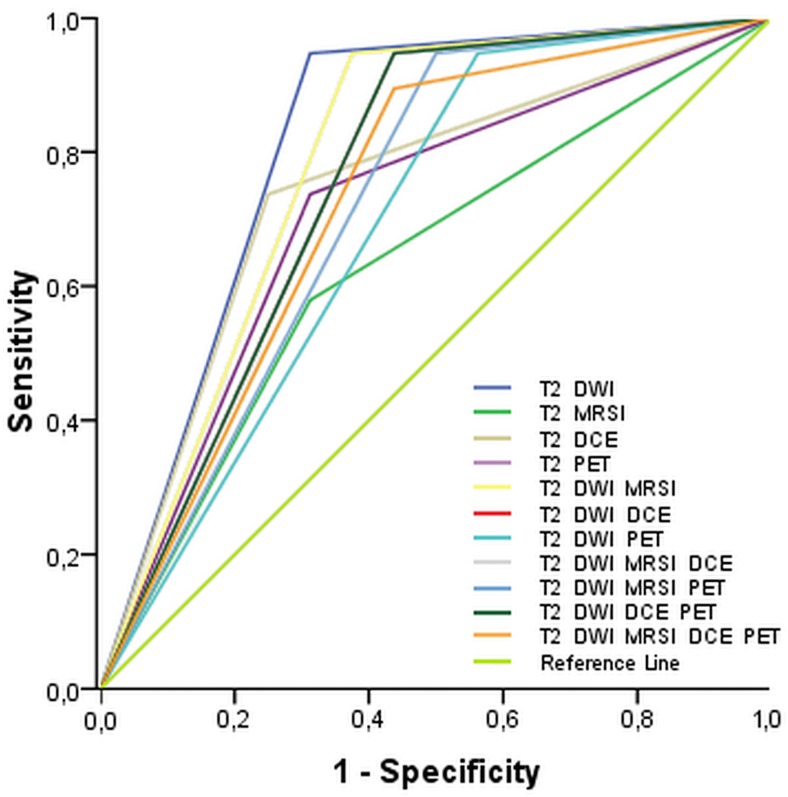
ROC curves for all lesions of the prostate independent of the Gleason score depict the diagnostic accuracy of all investigated MP reading approaches.

The ROC analysis in Fig 2 illustrates the diagnostic accuracies of each single parameter and all assessed MP imaging combinations for the detection of PCa. MP imaging with the two MRI parameters T2w and DWI yielded the highest AUC (0.82) compared to the other combinations, with AUCs ranging from 0.54 to 0.78.

There were four false-negatives [one low grade Gleason 6 (3+3) tumor; one intermediate grade Gleason 7 (4+3)] tumors on MP [^11^C]Acetate PET-MRI with absent focal uptake. On MP MRI with T2 and DWI there were only two false-negatives, [one Gleason 6 (3+3) tumor; one Gleason 8 (4+4)]. Both were very small lesions (<0.5cm), and thus, not visible on MP MRI. All false-positives, either with MP MRI with T2 and DWI (n = 5), or MP [^11^C]Acetate PET-MRI (n = 7), comprised BHP or prostatitis.

Detailed histopathologic results for all false-positive and false-negative lesions for [^11^C]Acetate PET-MRI and T2w-MRI with one, two, three, or four additional parameters, and all single parameters, are provided in Supporting Information S1 Table.

### Distant staging

In 15 patients with radical prostatectomy and pelvic LN dissection, 60 LN regions were evaluated. Eleven were found to be positive in histopathological evaluation (positivity rate, PR = 18.3%). MP MRI rated eight regions in six patients positive for regional LN metastases, which was confirmed by histopathology. In three regions in two patients, MP MRI was rated false-negative, resulting in a sensitivity, specificity, and diagnostic accuracy for MP MRI of 72.7%, 100%, and 95%, respectively.

[^11^C]Acetate PET /CT rated 13 regions in nine patients positive for regional LN, which was confirmed by histopathology. [^11^C]Acetate PET/CT did not miss any LN metastases, but, in one patient, two regions were rated false-positive. MP [^11^C]Acetate PET-MRI had an improved sensitivity, specificity, and diagnostic accuracy for the detection of regional LN metastasis of 100%, 96%, and 97%, compared to MRI or PET

In one patient, MP [^11^C]Acetate PET-MRI could identify a liver metastasis that could not be detected by MP MRI alone, as it was out of the dedicated pelvic MRI field of view. In addition, both MP MRI and MP [^11^C]Acetate PET-MRI correctly detected osseous metastases in four patients. In our patient cohort no additional distant lymph node metastases were identified.

## Discussion

This study aimed to demonstrate the feasibility of MP [^11^C]Acetate PET-MRI for the non-invasive quantitative assessment of multiple hallmarks of cancer, such as tumor neoangiogenesis, cell-membrane turnover, fatty acid synthesis, and microstructural and cellular changes, and to investigate the value of the individual MRI and PET parameters, as well as their combinations. The results of our study show that MP [^11^C]Acetate PET-MRI merges morphologic with functional information, and allows insights into the molecular and metabolic processes involved in cancer development. The combination of two MRI-derived parameters (T2 and DWI) yields the highest diagnostic accuracy. MP [^11^C]Acetate PET-MRI facilitates an improved distant staging of regional lymph nodes and identifies organ and bone metastases, providing “one -stop” staging in patients with primary PCa.

In this study for the diagnosis of primary PCa, the combination of the two MRI parameters T2w and DWI yields the highest sensitivity, specificity, and diagnostic accuracy (95%; 69%; 88%), with an AUC of 0.891. The other MRI parameters (DCE, MRSI) and [^11^C]Acetate PET alone show a limited sensitivity, specificity, and diagnostic accuracy, but it should be noted that they are not intended as stand-alone parameters [18]. Similar results were found in previous studies investigating MP MRI of the prostate using either three or four MRI parameters [19–22] and consequently in the new version of the PI-RADS (V2) MRSI is no longer included as such in the evaluation process. However, there are several studies that demonstrate, in a head to head comparison of PI-RADS V1 and V2, independent of the zonal location, that PIRADS V1 performs better than PIRADS V2 [23, 24]. Furthermore, previous studies have shown that the PI-RADS V1 is reliable and reproducible when using a sum score weighting each sequence equally [24–27] and therefore our analysis is based on PI-RADS V1 as we aimed to exploit the full potential of MP MRI by using all available imaging parameters. The PI-RADS guidelines are a “living document” and due to ongoing research more changes over the time can be expected.

PET offers unique functional information on cancer hallmarks, and has been demonstrated to add valuable information in the assessment of different cancers [28, 29]. In this context, we evaluated PET with the radiotracer [^11^C]Acetate as the fifth parameter. MP [^11^C]Acetate PET-MRI was beneficial for distant and local staging, but not in the detection of primary localized PCa. The number of false-positive and false-negatives readings was similar to previous MRI or PET/CT studies [5, 19]. In addition to [^11^C]Acetate other radiotracers have been investigated in prostate cancer. Buchegger et al. found that a three-phase [^18^F]Choline PET/CT and an analogous [^11^C]Acetate PET/CT protocol showed a similar performance for early recurrent PCa staging on a per patient and a per lesion-basis [30]. Different to our patient cohort, the authors investigated patients with local recurrence of prostate cancer after therapy. Nevertheless, for distant staging the results are in good concordance with our findings confirming that both tracers are a valuable tool for the detection of distant metastases. Similar results were also reported by Kotzerke et al. who reported no difference between [^11^C]Acetate and [^11^C]Choline in the detection of PCA and its metastases [31].

There is agreement that both radiolabeled [^11^C]Acetate and [^11^C]/ [^18^F]Choline can influence patient management by detection of local recurrence, lymph node, or bone metastases of PCA. In a recent study, Lamanna et. al. investigated the intra-individual performance of [^18^F]Choline and [^11^C]Acetate PET/CT for restaging of recurrent PCA and its impact on patient managment by correlating the PET findings with long-term clinical and imaging follow-up [32]. The authors concluded that treatment approaches were influenced by [^11^C]Acetate or [^18^F]Choline PET studies in one third of the patients. This is in good agreement with the results of the current study, where distant lymph and bone metastases could not be identified by MRI alone but were detected by [^11^C]Acetate PET.

Despite the limited benefit of adding more functional parameters to the examination protocol for primary cancer detection, there is a rationale for MP [^11^C]Acetate PET-MRI using several functional parameters. MP MRI has been proven to aid in the selection of PCa patients and in monitoring during active surveillance [33]. In patients with advanced PCa treated with primary radiation therapy (RT), MP imaging-derived parameters are predictive of outcome and aid the definition of RT target/boost volumes [34–37]. Both PET and MP MRI are valuable in the detection of PCa recurrence [38–41], and initial results hint at the potential of MP PET-MRI in this context [42].

Recently novel targeted radiotracers that provide further insights into tumor biology are being translated from the preclinical to clinical imaging. Initial clinical studies using the radiotracer ^68^Gallium Prostate-Specific Membrane Antigen (^68^Ga PSMA) have shown excellent results results initiating its implementation in the work-up of prostate cancer patients [5, 43–46]. Other receptors overexpressed in prostate cancer can be targeted by specific radiolabeled imaging probes, e.g androgen receptors and gastrin-releasing peptide receptors and are under investigation [5]. It can therefore be assumed that the full potential of MP PET-MRI has not been fully realized and this study has also to be seen as a blue-print for future MP PET-MRI studies in prostate as well as other cancers.

Compared to MRI alone, MP [^11^C]Acetate PET-MRI improves the detection of regional LN metastases, which were not visualized due to the limited MRI field of view, but by PET being performed as a whole body examination. MP [^11^C]Acetate PET-MRI accurately detects organ and bone metastases. In addition to an accurate PCa detection, MP [^11^C]Acetate PET-MRI provides an improved “one-stop” local and distant staging. This influences treatment-planning strategies for patients undergoing either surgical procedures or radiation therapy thus enabling optimal therapeutic outcomes. Since it has been proven that in over 90% of relapsing PCa the recurrence occur in the location of the primary lesion [47–49], an accurate definition of DIL boost volumes are essential for patient outcomes and facilitated by functional imaging methods such as MP [^11^C]Acetate PET-MRI (“dose painting”) [50].

The current study has several limitations. First, not all patients underwent MP [^11^C]Acetate PET-MRI on the same day (average 2.9d; median 0d; max = 14d). Nevertheless, the vast majority (90%) of examinations were performed on the same day and thus no relevant tumor microenvironment changes were expected that might have altered the results. [^11^C]Acetate PET-MRI performed fused and not simultaneously. However, all potential spatial mis-registration could be accounted for using dedicated software. Moreover, the above-mentioned limitation can be circumvented by the use of hybrid PET-MRI scanners, which are now clinically available. Second, the histopathological lymph node verification was just possible in 15 out of 56 patients as not all patients underwent radical prostatectomy. There were several patients with a low grade carcinoma in whom radiotherapy and active surveillance were often the therapy of choice.

In conclusion, the results of our study demonstrate that MP [^11^C]Acetate PET-MRI enables insights into tumor biology. MP [^11^C]Acetate PET-MRI with two parameters (T2 and DWI) yields the highest diagnostic accuracy. MP [^11^C]Acetate PET-MRI facilitates an improved local and distant staging, providing “one-stop” staging in patients with primary PCa, and therefore has the potential to improve therapy.

## Supporting information

S1 Fig MP [^11^C]Acetate PET-MRI performed in a 72-year-old patient with an elevated prostate-specific antigen (PSA) level (7.2ng/ml) at the time of imaging. (a) Axial 3-mm thick T2-w image (TR/TE/TI 4000/101/230ms) of the middle third of the prostate. The readers described a well-circumscribed hypointense lesion in the central zone (T2w-negative). (b) On the ADC map, the lesion presents as a focal area with low signal intensity, with corresponding high signal intensity on b800s/mm2 images (DWI-positive). (c-d) ^1^H-MRSI shows an elevated choline/citrate ratio in the suspicious region (^1^H-MRSI-positive). (e-f) The DCE-MRI shows a focal contrast enhancement for the suspicious area (e –T1w image 80s post contrast, f—K^trans^ map overlaid on T2w image) (DCE-positive). (g) [^11^C]Acetate PET-MRI shows a tracer hotspot in this area, with a maximal SUV of 6.5 (PET-MRI-positive). MP [^11^C]Acetate PET-MRI was rated false-positive in this patient. Histopathology obtained by image-guided biopsy showed a benign prostate hyperplasia.
(TIFF)

S1 Table Detailed histopathological results of false-negative and false-positive lesions. (PDF)
