# Supplementary figures and images for: Multiparametric [11C]Acetate positron emission tomography-magnetic resonance imaging in the assessment and staging of prostate cancer
Source: PLoS One. 2017 Jul 18;12(7):e0180790. doi: 10.1371/journal.pone.0180790 (PMC5515396; doi:10.1371/journal.pone.0180790)

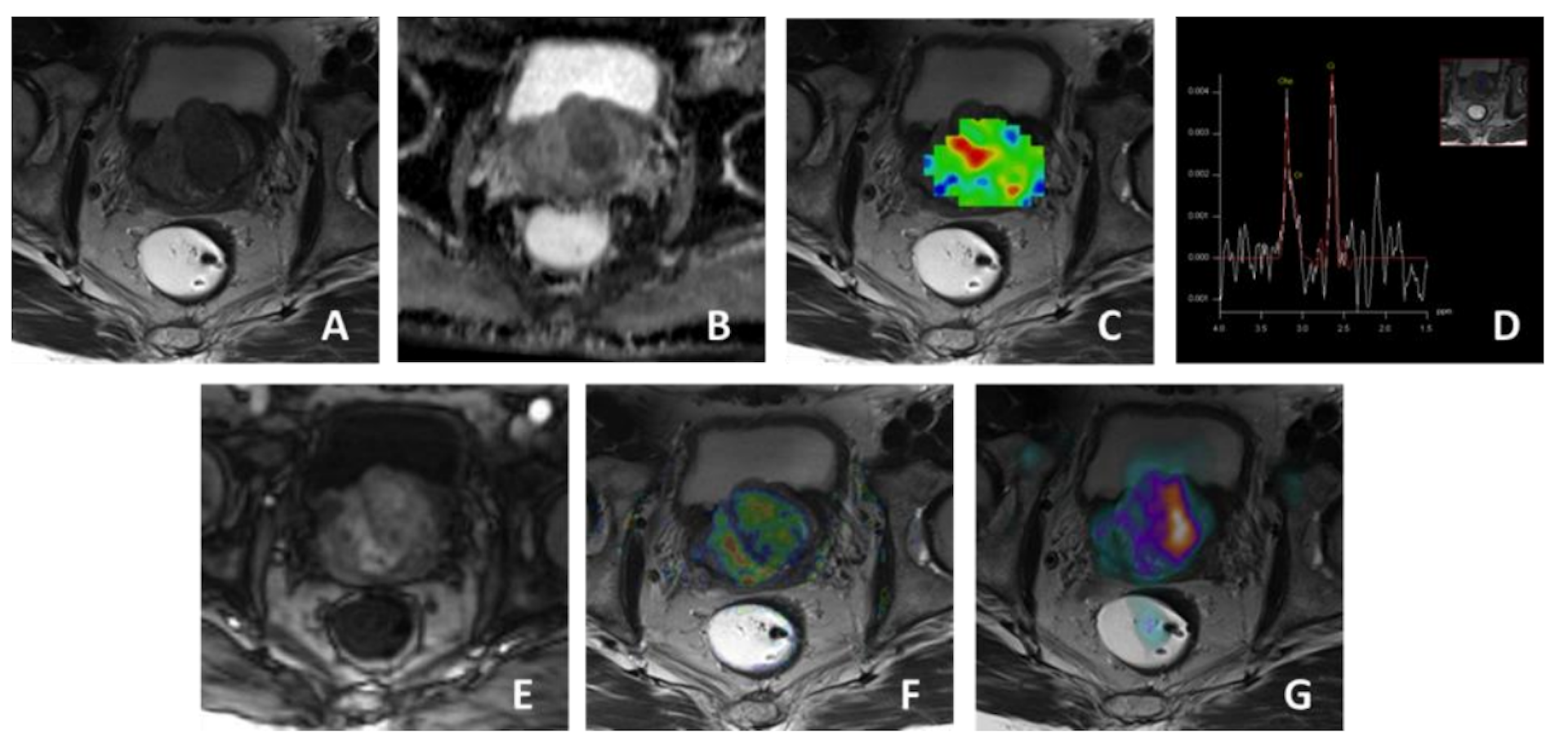

Supplement: S1 Fig — (a) Axial 3-mm thick T2-w image (TR/TE/TI 4000/101/230ms) of the middle third of the prostate. The readers described a well-circumscribed hypointense lesion in the central zone (T2w-negative). (b) On the ADC map, the lesion presents as a focal area with low signal intensity, with corresponding high signal intensity on b800s/mm2 images (DWI-positive). (c-d) 1H-MRSI shows an elevated choline/citrate ratio in the suspicious region (1H-MRSI-positive). (e-f) The DCE-MRI shows a focal contrast enhancement for the suspicious area (e –T1w image 80s post contrast, f—Ktrans map overlaid on T2w image) (DCE-positive). (g) [11C]Acetate PET-MRI shows a tracer hotspot in this area, with a maximal SUV of 6.5 (PET-MRI-positive). MP [11C]Acetate PET-MRI was rated false-positive in this patient. Histopathology obtained by image-guided biopsy showed a benign prostate hyperplasia. (TIFF) [file pone.0180790.s001.tiff]
