# Supplementary material for: Multiparametric [11C]Acetate positron emission tomography-magnetic resonance imaging in the assessment and staging of prostate cancer
Source: PLoS One. 2017 Jul 18;12(7):e0180790. doi: 10.1371/journal.pone.0180790 (PMC5515396; doi:10.1371/journal.pone.0180790)
Supplement: S1 Table — (PDF) [file pone.0180790.s002.pdf]

S1 Table: Detailed histopathological results of false-negative and false-positive lesions

	Imaging modality	False positives	*n*	False negatives	*n*
**Single parameter**	**T2w[1]**	3 Prostatitis<br>4 BPH[6]	***7***	1 GS 6 (3+3)<br>1 GS 8 (4+4)	***2***
	**DWI[2]**	4 Prostatitis<br>5 BPH	***9***	1 GS 6 (3+3)<br>1 GS 8 (4+4)	***2***
	**3D ^{1}H-MRSI[3]**	4 Prostatitis<br>3 BPH	***7***	8 GS 6 (3+3)<br>5 GS 7 (4+3)<br>1 GS 8 (4+4)	***14***
	**DCE[4]**	4 Prostatitis<br>3 BPH	***7***	5 GS 6 (3+3)<br>3 GS 7 (4+3<br>1 GS 8 (4+4)	***9***
	**PET[5]**	5 Prostatitis<br>6 BPH	***11***	4 GS 6 (3+3)<br>4 GS 7 (4+3<br>1 GS 8 (4+4)	***9***
**Two parameters**	**T2w and DWI**	3 Prostatitis<br>2 BPH	***5***	1 GS 6 (3+3)<br>1 GS 8 (4+4)	***2***
	**T2w and ^{1}H-MRSI**	2 Prostatitis<br>3 BPH	***5***	8 GS 6 (3+3)<br>5 GS 7 (4+3)<br>1 GS 8 (4+4)	***14***
	**T2w and DCE**	2 Prostatits<br>2 BPH	***4***	4 GS 6 (3+3)<br>4 GS 7 (4+3)<br>1 GS 8 (4+4)	***9***
	**T2w and PET**	3 Prostatitis	***5***	6 GS 6 (3+3)<br>4 GS 7 (4+3)	***11***

		2 BPH			
				1 GS 8 (4+4)	
**Three parameters**	**T2w, DWI and ^{1}H-MRSI**	3 Prostatitis	***6***	1 GS 6 (3+3)	***2***
		3 BPH		1 GS 8 (4+4)	
	**T2w, DWI and DCE**	3 Prostatitis	***7***	1 GS 6 (3+3)	***2***
		4 BPH		1 GS 8 (4+4)	
	**T2w, DWI and PET**	4 Prostatitis	***9***	1 GS 6 (3+3)	***2***
		5 BPH		1 GS 8 (4+4)	
**Four parameters**	**T2w, DWI, ^{1}H-MRSI and DCE**	3 Prostatitis	***7***	1 GS 6 (3+3)	***2***
		4 BPH		1 GS 8 (4+4)	
	**T2w, DWI, ^{1}H-MRSI and PET**	4 Prostatitis	***8***	1 GS 6 (3+3)	***2***
		4 BPH		1 GS 8 (4+4)	
	**T2w, DWI, DCE and PET**	3 Prostatitis	***7***	1 GS 6 (3+3)	***2***
		4 BPH		1 GS 8 (4+4)	
**MP $[^{11}C]$Acetate PET-MRI**	**T2w, DWI, ^{1}H-MRSI, DCE and PET**	3 Prostatitis	***7***	2 GS 6 (3+3)	***4***
				1 GS 7 (4+3)	
		4 BPH		1 GS 8 (4+4)	

[1] T2-weighted MRI

[2] diffusion-weighted imaging

[3] three-dimensional proton MR spectroscopic imaging

[4] dynamic contrast-enhanced MRI

[5] positron emission tomography

[6] benign prostatic hyperplasia
